# On Pure Oxide of Carbon, Considered as a Poison

**Published:** 1855-03

**Authors:** Adrien Chenol


*On Pure Oxide of Carbon, considered as a Poison.* By M. ADRIEN CHENOL.—The facts we are about to communicate relative to the poisonous action of pure oxide of carbon, lead to the following theory:—

As carbonic acid only plays the part of an *obturator,* and as, being a gas undecomposable at a low temperature, it is incapable of furnishing the oxygen necessary for the combustions which sustain life; it causes death by pure and simple asphyxia; it is quite otherwise with pure oxide of carbon.

This oxide of carbon, indeed, in contact with the marvellous organs of combustion with which we are endowed, speedily gives rise to the following effects:—It passes to the state of carbonic acid, whence result:—1. Substraction of oxygen and its consequences; 2. combustion of this oxygen and its consequences; 3. formation of carbonic acid and its consequences.

These three effects are inseparable, and the last occasions immediate asphyxia by arresting the action of the lungs and every act of motion. But at the same time, the oxygen has been condensed: whence has résulted an action of compression and of tearing by the vacuum produced; but, moreover, the conversion of oxide of carbon into carbonic acid has given rise to a disengagement of about 6000 calores per litre of oxygen, burned in the spongiosities of our organisation containing this oxygen, which should have served for enriching the blood.

These 6000 calores, developed in intimate contact and cellules of atoms or small spheres of oxygen, occasion, then, virtually and infallibly, a disorganisation by cauterisation, which causes that violent pain accompanying poisoning with *oxide of carbon*, which differs in this respect from that produced by *carbonic acid*, which, as we have frequently experienced in the mines of Pont-Gebaud, causes an agreeable intoxication, passing by degrees to a sweet lethargy, rather than a painful sensation. Consequently, the study of oxide of carbon as a poison is a subject of much complexity, and of the highest interest in a toxicological point of view.

*Facts in support of this Theory.*—Contrary to what has been written, the oxide of iron of ordinary combustion is an insufficient reducer, and incapable of removing oxygen from metals of the class of iron. Thus, the theories based on this action are erroneous. This is not the occasion for a discussion of this order. We may here say that this oxide of carbon presents dangers as a poison.

But if the oxide of carbon of combustion, that is to say, that which contains 4, 5, and even 6 volumes of nitrogen, is not a powerful reducer, pure oxide of carbon is not only a reducer of the greatest energy, but a violent poison, even in very feeble doses.

My health has suffered seriously, in consequence of several poisonings by this gas, of which I have made very frequent use, on account of its energetic and peculiar properties as a reducer. I wish, therefore, to obviate, for those who shall follow up my works, the dangers attached to their practice, by pointing out to them this danger in an especial manner.

In 1846, I was at the Marquis of Sassenay's manufactory, at Stolburg, in Prussia, for the purpose of studying the ores of zinc, and their treatment in various points of view, particularly as regards improving the quality of very poor ores, by a system which I had invented, and which consisted in fusing these poor ores in a *cubilat,* closed at the upper part, and collecting the zinc at different heights in various states of oxidation. In the course of my experiments, I desired to make some investigations concerning the oxide of carbon resulting from the reaction of the oxides mixed with an excess of charcoal. With this view, having no instrument suitable for collecting this gas, I drew it by means of a pipette from the apparatus, and conveyed it under a bell-glass. While I was occupied with this operation, the engineer of the establishment spoke to me, and struck me on the shoulder, without attracting my attention; I then, as it appears, inhaled some of the gas contained in my mouth, and immediately fell on my back, as if stunned.

The following were the external and internal effects of this sudden annihilation of all external faculties described, according to a *procès-vèrbal*, drawn up by the director, the engineer, and myself, after I had somewhat recovered; and this extract is perfectly in accordance with another accident, of which I will speak presently, and which occurred to me two months ago.

*Externally*:—1, I fell as if struck by lightning; 2, the eyes were turned back in their orbits; 3, the limbs were contracted; 4, the skin was discolored; 5, the veins were swelled, and presented a black tint under the skin. *Internally*:—1, The sensibility was extreme; life was, so to speak, exalted; all the ideas and principal matters of interest, and all the predominant affections were reproduced to the mind, as in an instantaneous mirror. 2. Violent pains, as if of tearing, were experienced in the thorax. This inward pain was most vivid; the brain felt powerfully compressed, either as a principal action, or as a nervous action, induced by the pain.

Being in this position, I was carried into the air without knowing it, nor did I feel the lotions of water and of vinegar, or the inhalations of ammonia, &c. After a quarter of an hour, external sensation slowly and progressively returned, accompanied by the internal pains already mentioned; but then changing into a sensation of suffocation, attended with cold, and with drops of perspiration all over the body, but especially on the head.

For several days the lassitude was general and continuous, and digestion very bad. Moreover, there was a general disgust for everything. Sleep, from being light, became obstinate and heavy; it was frequently disturbed by cramps in the knees and toes.

For several months after, these effects, although they diminished very much, maintained a marked influence on my health. I was inclined to sadness, faintness, and disgust:—I was alarmed at the noise of any unexpected shock; it produced a nervous shock analogous to an electrical discharge. This state gradually became modified, passing to that of a kind of insensibility which was fixed more particularly at the ends of my fingers, in degrees of intensity varying with the state of the atmosphere. To sum up, it is evident that poisoning with pure oxide of carbon is most terrible in itself, and involves a series of disorganisation.

I am now under the influence of the second poisoning to which I have alluded, and which happened through the sudden breaking of a manometer tube, against which I knocked my head in a narrow place. All the internal symptoms were exactly the same, but I was not knocked down. I remained in a state of half consciousness and capable of taking care of myself, according to what I hoped to do, either for others or for myself.

Feeling convinced that the internal derangement resulted from a lesion produced either by the effect which I have just mentioned as regards the theory of the poisonous action of oxide of carbon, I drank for several days, and in as large quantity as I could, gum water and mallowa water. I expected to attain my object as soon as possible. Nevertheless, the disgust, feebleness, and insurmountable inertness continually prevailed; the insensibility of the ends of the fingers became extreme; and, by a singular contrariety, not only did shocks cause me to tremble electrically, but a drop of water which fell on my skin, or the slightest touch, even my own, produced a sense of irritation. Bathing seemed to do me much good; for several hours after, the *calm* was much ameliorated; but sometimes in the baths, I experienced a kind of general shuddering.

I have addressed these details to the Academy for the sake of general utility, and in order that the medical profession may study the dangers of so terrible a poison, and the curative means to be employed.—*London Chemist, from Comptes Rendus.* No. 16.

